# 

**Published:** 1919-05-10

**Authors:** 


### American Optimism.

President George E. Vincent, of the Rockefeller Foundation for medical research, is reported as defining the 1919 programme of his body as the "elimination" of yellow fever, tuberculosis, malaria, and hookworm disease. The Foundation is to assist in the care of soldiers mentally and nervously disabled, and to spend £200,000 to develop a medical school at the University of Chicago. We do not doubt that there will be plenty of money to spend; indeed, the same report quotes the available funds at one-and-a-third millions sterling. But we do doubt the "elimination" of tuberculosis. Material resources do not in practice count for much more in really original research than they do in the fine arts. For instance, take the activities of this same association, which up to date have been directed mainly against tropical disorders. Yet it has accomplished nothing at all to rank with the discoveries of Manson, a general practitioner in China, or Ross, an obscure junior officer in the Indian Medical Service, who probably had not as many farthings to spend on their work as the Rockefeller Foundation has scores of pounds. However, complete success may follow this large outlay, and if so it will amply repay the expense.

